# Leguminous cover crops and soya increased soil fungal diversity and suppressed pathotrophs caused by continuous cereal cropping

**DOI:** 10.3389/fmicb.2022.993214

**Published:** 2022-10-06

**Authors:** Shuting Yu, Tianshu Wang, Yili Meng, Shuihong Yao, Li Wang, Haotian Zheng, Yanzheng Zhou, Zewei Song, Bin Zhang

**Affiliations:** ^1^National Engineering Research Center of Arable Land Protection, Institute of Agricultural Resources and Regional Planning, Chinese Academy of Agricultural Sciences, Beijing, China; ^2^College of Resources and Environment, Huazhong Agricultural University, Wuhan, China; ^3^College of Life Sciences, University of Chinese Academy of Sciences, Beijing, China; ^4^Economic Crops Institute, Jining Academy of Agricultural Sciences, Jining, China; ^5^BGI-Shenzhen, Shenzhen, China

**Keywords:** cover crop, legume–cereal rotation, soil fungal communities, symbiotroph, saprotroph, pathotroph

## Abstract

The enrichment of soil-borne fungal pathogens and a high input of mineral fertilizer in the continuous cropping of cereal crops have raised a concern about soil health deterioration. Conversion of continuous cereal cropping to a legume-involved system alters the soil fungal community. However, when a leguminous cover crop is grown with a succeeding legume grain crop such as soya (*Glycine max L. Merril*), the effects on the soil fungal community when two legumes are involved in the crop system remain unclear. Thus, the effects of the cover crop on the soil fungal community under a succession of soya and a succession of maize (*Zea mays L.*) were clarified: a continuous wheat (*Triticum aestivum L.*)–maize cropping system was converted to new rotation systems with three cover crop treatments: leguminous vetch (*Vicia sativa L.*), a mixture of vetch and rye (*Secale cereale L.*), and fallow, succeeded by soya or maize in this study. The soil fungal community at the harvest of soya and maize were determined using high-throughput sequencing of ITS2 amplicons. Compared to a wheat–maize rotation system, all of the new rotation systems that involved leguminous crops or fallow increased the soil fungal diversity and suppressed pathotrophs by reducing the soil NH_4_^+^, NO_3_^−^, available K, and available P concentrations. Different cover crops changed the fungal community composition, but their effect was overwhelmed by the strong effect of succeeding soya, which induced minor shifts among the cover crop treatments under soya than maize. The Vetch–Soya system exhibited the highest fungal diversity, which have been due to an increase of symbiotrophs. Replacing wheat with mixed vetch and rye most greatly suppressed the pathotrophs, and this suppression effect was stronger when succeeded by maize than by soya. These results showed the short-term benefits of legume–legume succession and legume–cereal mixed cover crops for increasing fungal diversity and suppressing pathotrophs. Further study is needed to examine the long-term effects of Vetch–Soya on the accumulation of legume-associated pathogens.

## Introduction

Fungi are the major component of the soil microbial biomass (Högberg et al., 2002; Joergensen and Wichern, 2008). They play an important role in controlling plant litter decomposition (Gil-Martínez et al., 2021), nutrient cycling and acquisition (Ai et al., 2018; Cloutier et al., 2020), plant diseases (Bainard et al., 2017; Liu et al., 2019), and soil aggregate formation (Six et al., 2006). Cropping systems can affect the soil fungal diversity and community by changing the C input from root exudates, plant litter decomposition, and mineral fertilization, as well as their impact on the availability of soil water and nutrients (Kim et al., 2020; Muhammad et al., 2021). Continuous cropping of cereal crops adds a large amount of high C/N straw and requires much mineral N fertilizer that can enrich plant residue-borne pathogens, such as *Gaeumannomyces graminis* var. *tritici*, *Fusarium graminearum*, and *Fusarium moniliforme*, as well as symbiotrophs such as *Arbuscular mycorrhiza* (Cook, 2003; Miedaner and Juroszek, 2021). In the North China Plain, decades of continuous wheat–maize cropping coupled with a high input of mineral fertilizer have raised concern about soil health and environmental deterioration (Bei et al., 2018; Saeed et al., 2022). Introducing legume crops into cropping systems could be an effective way to control fungal pathotrophs and reduce mineral fertilization.

Legumes are increasingly used as cover crops and rotated with high N-demanding cereal crops such as maize (*Zea mays L.*). As a cover crop, a legume can improve the yield of the succeeding cereal crop and soil health by reducing soil erosion, increasing soil water and nutrient availability, and controlling soil-borne pathogens (Cook, 2003; Detheridge et al., 2016). Legume cover crops suppress pathogens and increase the soil fungal diversity by reducing the fertilizer input and adding other type of plant residues (Esmaeilzadeh-Salestani et al., 2021). Moreover, mixed cover crops that contain multiple species of plants further increase soil fungal saprotrophs due to a better C/N ratio and a higher biomass of plant litter than a single-species cover crop (Borrell et al., 2016; Detheridge et al., 2016; Bainard et al., 2017). As a grain crop, leguminous soya (*Glycine max L. Merril*) rotated with a cereal crop can maintain sustainable crop production and significantly increase the soil microbial biomass (Higashi et al., 2014; Chavarría et al., 2016; Gong et al., 2021). Legume–cereal rotation increases the diversity of organic C inputs from plant litter and root exudates (Thapa et al., 2021). A more diverse system has been found to recruit beneficial fungi, e.g., *Mortierella* and *Paecilomyces* (Hontoria et al., 2019; Liu et al., 2020), and can break the life cycle of fungal pathotrophs (Uzoh et al., 2019; Chamberlain et al., 2021).

The benefits of legume-involved systems have been demonstrated in many studies; however, systems with legumes may also have some potential detrimental effects. Specifically, when a leguminous cover crop is grown with a succeeding legume, the effects on the soil fungal community when two legumes are involved in the crop system remain unclear. Continuous cropping of legumes also has been found to increase soil pathogens, such as *Thanatephorus*, *Fusarium*, *Alternaria*, and *Clonostachys* (Liu et al., 2019, 2020). Biological N-fixation can decrease the soil pH, which may accelerate soil acidification. While legume crops demand less N, more phosphorous (P) and potassium (K) must be acquired from the soil, which changes the balance of soil nutrients and shifts the fungal community composition (Haynes, 1983; Zhou et al., 2017). Since soya is the major grain crop in the North China Plain, an understanding of the impact on the soil fungal community composition of the addition of leguminous cover crops to a soya system, especially on soil-borne pathogens, is crucial. In addition, the effects of the same leguminous cover crop species on a soya system should be compared to a maize system.

Accordingly, a field experiment was undertaken to convert a prior wheat–maize rotation system to a new cropping system involving leguminous crops or winter fallow, which included hairy vetch (*Vicia sativa L.*) mixed with the cereal crop rye (*Secale cereale L.*) as cover crops, succeeding soya and maize as grain crops. The objectives of this study were (1) to determine the short-term effects of converting the prior wheat–maize cropping system to the new legume-involved crop rotation systems on the soil fungal diversity and community composition, and (2) to compare the effects of the cover crop on the soil fungal community under a succession of maize and soya. It was hypothesized that (1) the soil fungal diversity and community composition in the systems with leguminous cover crops would differ from that of wheat–maize system; (2) a more diverse cropping system would recruit more saprotrophs and symbiotrophs and suppress pathotrophs, and the cover crop effect would be different under succeeding maize than under succeeding soya.

## Materials and methods

### Experimental site

The field experiment was established in October 2017 at the experimental station of the Farmland Irrigation Research Institute, Chinese Academy of Agricultural Sciences, Xinxiang City of Henan Province (35°19′ N, 113°53′ E) in China. The area has a typical warm temperate continental monsoon climate, with a mean annual precipitation of 573.4 mm and a mean annual air temperature of 14.0°C. The soil was a sandy loam (701 g kg^−1^ sand, 141 g kg^−1^ silt, 157 g kg^−1^ clay). For the four decades prior to the experiment, the cropping system was wheat followed by maize and regular chemical fertilizers were applied. Winter wheat was grown from the middle of October to early June, with 255 kg N ha^−1^, 112.5 kg P_2_O_5_ ha^−1^, and 75 kg K_2_O ha^−1^ applied. Summer maize was grown from early June to early October, with 180 kg N ha^−1^, 90 kg P_2_O_5_ ha^−1^ and 120 kg K_2_O ha^−1^ applied.

### Experimental design

The field experiment was laid out in a completely randomized design with four replicates, and the plots were 8 × 6 m (Supplementary Figure S1). The cropping system treatments included three winter cover crop treatments: leguminous hairy vetch (*Vicia sativa L.*; Vetch), a mixture of vetch with cereal rye (*Secale cereale L.*; VetchRye), and fallow without a cover crop (Fallow), following two summer grain crops (Maize and Soya). In total, six treatments were employed in this study, designated as Fallow–Soya, Vetch–Soya, VetchRye–Soya, Fallow–Maize, Vetch–Maize and VetchRye–Maize.

Winter cover crops were sown on 15th October 2017 at a seeding rate of 90 kg ha^−1^ for Vetch, and 60 kg vetch ha^−1^ and 30 kg rye ha^−1^ for VetchRye. The above-ground biomass of winter cover crops in each plot was sampled at three randomly selected sites in an area of 0.25 m^2^ prior to chopping and incorporating them into the 10–15 cm soil layer using a rotary on 25th April 2018.

One week after incorporation, soya (cv. Jidou 17) and maize (cv. Zhengdan 958) were sown at 15200 soya plants ha^−1^ (40 cm between rows and 11 cm between plants in rows) or 60,000 maize plants ha^−1^ (60 cm between rows and 25 cm between plants in rows). No fertilizers and pesticides were applied to either winter or summer crops. Weeds were controlled manually in all plots. Soya plants suffered from the ever-green disorder (Hill et al., 2013), and no grain was harvested. On 3rd October 2018, the maize cobs were harvested by handpicking from two 6 m rows in the center of each plot to estimate maize grain yield. After that, all soya plants from five 6 m long rows in each plot were cut at the basal stem to estimate the aboveground biomass. The aboveground biomass of maize in each plot was calculated using a harvest index of 0.49 as reported in a study in the North China Plain (Wang et al., 2015). After drying at 70°C to constant weight, the soya plant biomass and maize grain yield were expressed as kg ha^−1^.

### Soil sampling and analysis

Soil samples were taken prior to the experiment from the 0 to 20 cm layer using an auger with an inner diameter of 3.5 cm from nine randomly selected sites on 10th October 2017 to make three composite soil samples. Soil samples after the experiment were taken using the same method from two sites between plants in each plot at the harvest of soya and maize on 3rd October 2018, resulting in 48 soil samples for each rotation system (3 cover crops × 2 grain crops × 4 replicates × 2 samples per plot).

The soil samples were stored in polyethylene bags with ice packs (4°C) and transferred to the laboratory. After removing the fine litter, root residue, and stones, part of the samples was ground to pass through a 2 mm sterilized sieve and stored at −20°C for DNA extraction. The others were used to measure the soil water content, soil pH and the content of organic carbon (C), nitrogen (N), phosphorous (P), and potassium (K). The soil water content was measured using an oven-drying method. Soil pH (soil to water 1: 2.5) was measured using a pH meter (Sartorius PB-10). Soil organic C and total N were measured using an elemental analyzer (Vario MACRO Cube, Elementar, Germany) after removing the inorganic C using a 1 M hydrochloric acid solution. Ammonia and nitrate were measured using a continuous flow autoanalyzer (Seal Auto Analyzer 3, Seal Analytical; Maynard et al., 1993). Available P and K were measured using the molybdenum-blue colorimetry and flame spectrophotometry methods, respectively (Olsen et al., 1954; Helmke et al., 1996).

### Soil DNA extraction and high-throughput sequencing

Soil total DNA was extracted from 0.5 g of soil using a NucleoSpin 96 Soil kit (MACHEREY-NAGEL) following the manufacturer’s instructions. The internal transcribed spacer 2 (ITS2) region of the fungal genes was amplified with the forward primer ITS7 (5′-GTGARTCATCRARTYTTTG-3′) and the reverse primer ITS4 (5′-CCTSCSCTTANTDATATGC-3′) with a 6 bp barcode at the 5′ end for sample splitting (Sun et al., 2021). Polymerase chain reaction (PCR) was performed under the conditions of 98°C for 5 min in a 25 μl mixture of 5 μl HiFi Buffer (5×), 1 μl deoxynucleotide triphosphates (dNTPs, 2.5 mM), 1 μl each primer (10 μM), 0.5 μl KAPA enzyme (5 U μl^−1^), 15.5 μl ddH_2_O, and 1 μl template DNA (20 ng μl^−1^), followed by 10 cycles at 98°C for 30 s, 50°C for 30 s, and 72°C for 1 min, and then 25 cycles at 98°C for 30 s, 52°C for 30 s, 72°C for 1 min, and a final extension at 72°C for 5 min. After amplification, the final DNA concentration and purification were determined using a Qubit 3 Fluorometer (Invitrogen). The DNA extraction quality was checked using 1% agarose gel electrophoresis. The purified PCR products were mixed at an equimolar ratio for sequencing using DNBSEQ-G400 (Emmanuel et al., 2010) with the paired-end 200 mode in BGI-Shenzhen.

### Analysis of sequencing data

All sequencing data were processed using a Python-based *Snakemake* pipeline (Sun et al., 2021). Primers and adapters were trimmed from pair-ended, demultiplexed fastq reads using *Cutadapt* (Martin, 2011). Low-quality reads with a maximum expected error rate larger than 1 and a minimum length less than 100 bp were discarded by Vsearch (Rognes et al., 2016). The sequencing of the fungal ITS2 region resulted in 30,627–126,187 sequences per sample after quality filtering. The sequences were grouped into different operational taxonomic units (OTUs) using a 97% similarity level by mode BEST (Edgar, 2010). OTUs with less than 10 total reads in all samples were removed. The standardized sequences of each fungal OTU were blasted against the UNITE (v7.2) database to designate taxonomic annotation (Abarenkov et al., 2010). To assess fungal diversity and community composition among the soil samples in a comparable manner, each soil sample was normalized to 10,000 sequences in the OTU table, resulting in 800 OTUs. Rarefaction curves of the observed richness (Supplementary Figure S2) were calculated in R.3.6.1^1^ using 1,000-fold resampling without replacement if the rarefied sequencing data represented most of their compositions (Zhong et al., 2015). FUNGuild v1.0^2^ was applied to classify the OTUs into eight functional guilds, i.e., pathotroph, saprotroph, symbiotroph, pathotroph–saprotroph, pathotroph–symbiotroph, saprotroph–symbiotroph, pathotroph–saprotroph–symbiotroph, and unidentified fungi (Nguyen et al., 2016). Based on the confidence ranking given by FUNGuild, the OTUs identified as highly probable were included directly, and OTUs identified as probable and possible were then manually checked using the Index Fungorum database^3^ and based on Gao et al. (2020). Finally, 287 genera were assigned to eight functional guilds (Supplementary Table S1), and the relative abundance was calculated for each rotation system.

### Statistical analysis

All statistical analyses and visualizations were performed in R. The Richness index refers to the observed number of OTUs in each sample (Fan et al., 2017), while the Shannon index was calculated from the richness matrix using the *diversity* function of the *vegan* package (Oksanen et al., 2013). A principal coordinate analysis (PCoA) was performed to determine the effects of the rotation system on the fungal community composition based on a Bray–Curtis distance matrix at the OTU and genus level, respectively (Gower, 1966). The significance of the effects was assessed using a permutational multivariate analysis of variance (PERMANOVA) with 999 random permutations (*p* ≤ 0.05), which was performed using the *adonis* function of the *vegan* package (Legendre and Caceres, 2013). Pair-wise comparisons of all rotation systems were conducted using a pairwise PERMANOVA implemented in the pairwise Adonis package.^4^ To identify the environmental factors (i.e., aboveground biomass and soil physicochemical properties) of the fungal community composition, all environmental factors were fitted to PCoA ordination plots using the *envfit* function of the *vegan* package, and significance was tested using a permutation test with 999 permutations (Lammel et al., 2018). A two-way ANOVA followed by Tukey’s HSD test in the *agricolae* package was performed to determine the effects of the winter cover crop and the grain crop on the aboveground biomass, soil properties, Richness index, Shannon index and the relative abundance of the classified phyla, genera and functional guilds at *p* ≤ 0.05. Only the genera with relative abundances >0.1% were compared by a two-way ANOVA. A functional fungal genus that was significantly different prior to and after the experiment, and among cover crops in new rotation systems was defined as a differential functional genus. A Random forest analysis (RFs) was performed using the *randomForest* function of the *randomForest* package to identify which environmental factors were the main drivers of the Richness index, Shannon index, and main functional guilds (pathotroph, saprotroph, and symbiotroph; Liaw and Wiener, 2002). In the RFs, environmental factors were included as predictors of the Richness index and Shannon index, and main functional guilds as response variables. The significance values of the cross-validated R^2^ and the whole model were examined using the *a3* function in the *A3* package. The significance of each predictor on the response variables was assessed with 5,000 response variable permutations using the *rfPermute* function of the *rfPermute* package. A Pearson correlation analysis was performed using the *rcorr* function of the *Hmisc* package to identify the significant correlations between differential genera and environmental factors (Strom et al., 2020).

## Results

### Aboveground plant biomass and soil physicochemical properties

In the wheat–maize rotation system prior to the experiment, the aboveground biomass of wheat was significantly higher than that of Vetch and VetchRye (*p* ≤ 0.001), with relatively high levels of soil available nutrients (Table 1). After the one-year experiment under the new rotation systems, soil pH, available P, and NO_3_^−^ contents of new rotation systems were significantly decreased (*p* ≤ 0.001), while the soil N/P, K/P, and NO_3_^−^/K were significantly increased (*p* ≤ 0.001).

**Table 1:** Aboveground biomass of cover and grain crops and the soil properties in systems prior to and after the experiment, and the *p*-values of the two-way analysis of variation (ANOVA) on the effects of cover crop, grain crop and their interaction in the new rotation systems.

	Cropping systems		Aboveground biomass		Soil properties											
			Cover crop	Grain crop	pH	SWC	SOC	TN	AK	AP	NH_4_^+^	NO_3_^−^	C/N	N/P	K/P	NO_3_^−^/K
				kg ha^−1^	g kg^−1^	mg kg^−1^										
Prior to experiment:	Wheat	Maize	8,474 a	19,855 a	8.65 a	145 a	9.6 a	1.2 a	150.0 a	42.5 a	4.1 a	26.3 a	7.8 a	28.9 d	3.5 e	5.7 d
New rotations:	VetchRye	Soya	3,044 b	4,192 d	8.45 cd	126 b	9.9 a	1.4 a	127.1 bc	15.5 d	3.9 ab	7.2 c	7.4 bc	85.9 bc	8.0 a	18.0 ab
	Vetch	Soya	2041 c	3,430 e	8.39 d	129 b	10.0 a	1.5 a	147.8 ab	14.9 e	3.4 bc	9.8 b	6.9 c	97.7 ab	9.9 a	15.2 bc
	Fallow	Soya	0 d	3,377 e	8.45 cd	134 b	9.3 a	1.3 a	139.1 b	13.6 f	3.1 c	11.0 b	6.8 c	101.4 a	10.2 b	12.8 c
	VetchRye	Maize	2,950 b	18,203 ab	8.49 bc	144 a	9.8 a	1.3 a	139.1 b	18.2 b	3.7 b	6.7 c	7.6 ab	75.3 c	7.7 d	21.5 a
	Vetch	Maize	2,198 c	17,900 ab	8.51 bc	144 a	9.7 a	1.3 a	119.3 c	17.0 c	3.5 bc	7.9 c	7.5 ab	72.3 bc	7.0 c	15.5 bc
	Fallow	Maize	0 d	16,460 b	8.56 b	129 b	9.5 a	1.2 a	101.8 d	15.2 de	3.0 c	5.2 d	7.7 ab	82.4 bc	6.7 bc	20.6 a
One-way ANOVA (*p*-value): effects of the cropping system conversion																
Cropping system conversion			**<0.001**	**0.03**	**<0.001**	0.08	0.92	0.41	0.07	**<0.001**	0.07	**<0.001**	0.23	**<0.001**	**<0.001**	**<0.001**
Two-way ANOVA (*p*-value): effects of cover crops and grain crops in new rotation systems																
Cover crop			**<0.001**	**0.008**	0.11	**0.04**	0.64	0.69	**0.006**	**<0.001**	**0.002**	**<0.001**	0.84	0.24	**0.02**	**<0.001**
Grain crop			0.43	**<0.001**	**< 0.001**	**<0.001**	0.93	0.26	**<0.001**	**<0.001**	0.68	**<0.001**	**0.04**	**0.004**	**<0.001**	**<0.001**
Cover crop × Grain crop			0.3	0.13	0.21	**<0.001**	0.89	0.68	**<0.001**	0.46	0.83	**<0.001**	0.23	0.7	**<0.001**	**0.01**

Lowercase letters in each column indicate significant differences among cropping systems at *p* ≤ 0.05. Bold numbers indicate significant effects at *p* ≤ 0.05. VetchRye is the mixture of vetch and rye. SWC, soil water content; SOC, soil organic carbon; TN, total nitrogen; AP, available phosphorus; AK, available potassium; C/N, the ratio of SOC and TN; N/P, TN, and AP; NO3^–^/K, the ratio of soil nitrate and AK.

Among the new rotation systems, VetchRye produced the highest above-ground biomass (2949.5 ~ 3044.4 kg ha^−1^), which was 34.2 ~ 49.2% higher than Vetch. Compared to the Fallow treatment, VetchRye significantly increased the plant biomass of succeeding Soya but not of Maize, while Vetch had no effect on the plant biomass of Soya or Maize. Soil water, the available K, and NO_3_^−^ contents, K/P, and NO_3_^−^/K were significantly affected by the cover crop, grain crop, and their interaction (*p* ≤ 0.05). Available P was affected by the cover crop and the grain crop (*p* ≤ 0.001), while soil NH_4_^+^ and pH were only affected by the cover crop and the grain crop (*p* ≤ 0.05), respectively. With the same cover crop, soil pH, water, and available P contents were lower under Soya than Maize. On the contrary, soil available K and NO_3_^−^ contents were higher under Soya than Maize, when the winter treatments were Vetch and Fallow. Soil available P and NH_4_^+^ contents were the highest in Fallow and the lowest in VetchRye followed by both Maize and Soya. The soil water and available K contents were the highest in VetchRye, and the soil water, available K and NO_3_^−^ contents were the lowest in Fallow followed by Maize. The soil NO_3_^−^ content was the lowest in VetchRye followed by Soya.

### Soil fungal diversity and community composition

**Figure 1:**
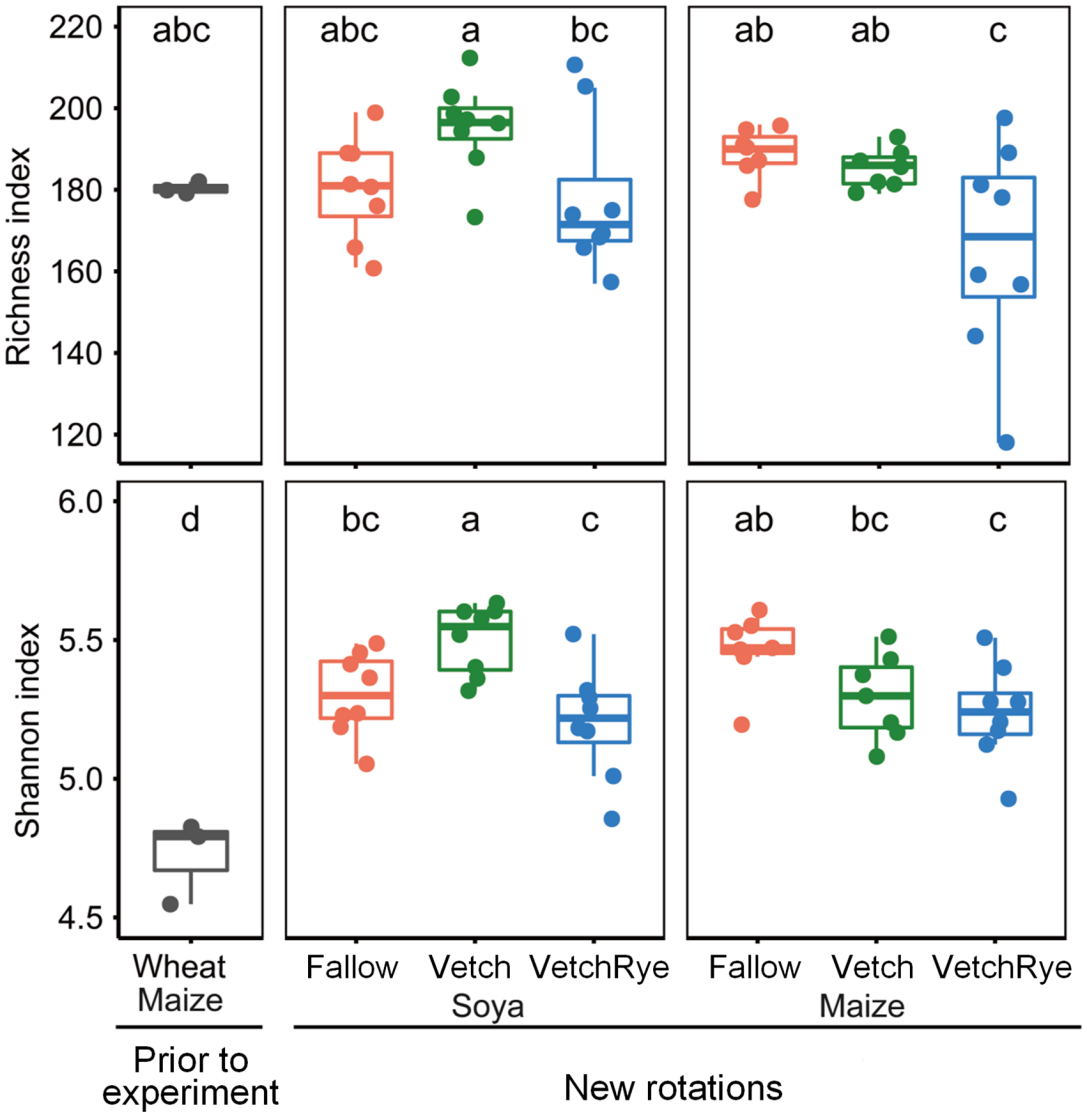
Richness and Shannon indices of the soil fungal community in the new rotation systems compared to those present prior to the experiment. Lowercase letters at the top of the boxes indicate significant differences among all cropping systems at *p* ≤ 0.05.

The average values of the fungal Richness and Shannon indices in the prior wheat–maize rotation system were 182 and 4.7, respectively, and changing the rotation system significantly (*p* ≤ 0.05) increased fungal Shannon index to an average of 5.3 (Figure 1). Of the new rotation systems, the Vetch–Soya system that included two leguminous crops had the highest fungal Richness and Shannon indices, while VechRye had the lowest fungal Richness and Shannon indices regardless of whether Soya or Maize was the succeeding crop.

**Figure 2:**
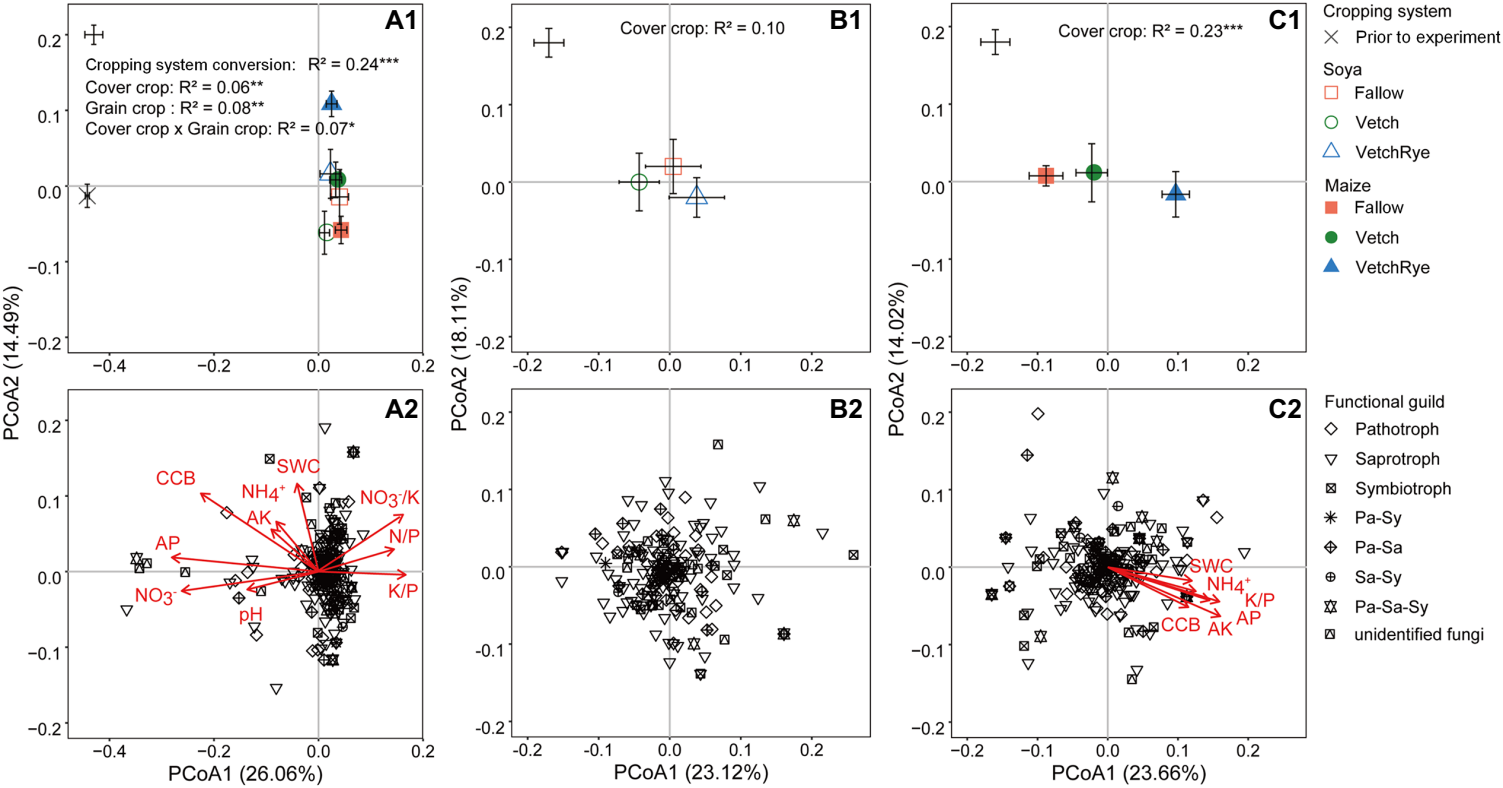
Principal coordinate analysis (PCoA) of the Bray–Curtis distances of fungal communities at the genus level **(A1–C1)**, the loadings of individual fungal genera assigned to different functional guilds and the factors affecting the PCoA ordinations **(A2–C2)**, showing the effect of cropping system conversion **(A1,A2)**, and cover crops followed by soya **(B1,B2)** and maize **(C1,C2)**. The average of rotation systems is presented as square markers ± standard error (prior to the experiment, *n* = 3; new rotation systems, *n* = 8). The asterisks following the determination coefficient (R^2^) indicate the significant differences at *p* ≤ 0.05 (*), *p* ≤ 0.01 (**) or *p* ≤ 0.001 (***) based on the permutational multivariate analysis of variance (PERMANOVA). CCB, cover crop biomass; Pa, pathotroph; Sa, saprotroph; Sy, symbiotroph. Abbreviations of soil properties are listed in Table 1.

The PCoA analysis showed differences in the fungal community composition between the prior and new cropping systems and among the new cropping systems, and the separation patterns were similar at the genus and OTU levels (Figure 2; Supplementary Figure S3). At the genus level, the composition of the soil fungal community was distinctly separated (*p* ≤ 0.001; detailed *p*-values of a pairwise comparison are presented in Supplementary Table S2) by the systems prior to and after the experiment, cover crop, and grain crop, and their interactions (*p* ≤ 0.05; Figure 2A1). The cover crop separated the fungal community composition more distinctly followed by Maize (*p* ≤ 0.001) than Soya (not significant; Figures 2B1,C1). The clear separation in the PCoA of the fungal community composition between the systems prior to and after the experiments, and among cover crops under Maize cropping, indicate selective enrichment of the fungal genera with certain functional guilds (Figures 2A2,C2). The separation prior to and after the experiment was primarily explained by PCoA1, which was associated with the variations of soil pH, available P, available K, NO_3_^−^, N/P, K/P, and K/NO_3_^−^, however, PCoA2 explained the separation among the new rotation systems, which was associated with variations of the soil water and NH_4_^+^ contents (Figure 2A2; Supplementary Table S3). Under Maize cropping, the separation among the cover crops was associated with the variations of cover crop biomass, NH_4_^+^, available P and K, and K/P (Figure 2C2; Supplementary Table S3).

The relative abundances of 6 phyla varied among the rotation systems (Table 2). Changing from the wheat–maize system to the new rotation systems increased Ascomycota and Basidiomycota and decreased Chytridiomycota and Undefined phyla. For the new rotation systems, the cover crop and the grain crop significantly affected the relative abundance of Basidiomycota and Mortierellomycota. However, VetchRye had opposite effects under Soya and Maize. VetchRye–Maize had the most abundant Mortierellomycota, while VetchRye–Soya had the least abundant Mortierellomycota among the new rotation systems. Moreover, the relative abundance of Basidiomycota was higher in VetchRye–Soya than VetchRye–Maize.

**Table 2:** Relative abundance of fungal phyla in the new rotation systems compared to those present prior to the experiment.

	Cropping systems		Relative abundances of fungal phylum %					
			Ascomycota	Basidiomycota	Mortierellomycota	Mucoromycota	Chytridiomycota	Unidentified
Prior to experiment:	Wheat	Maize	63.3 b	3.4 c	7.3 b	8.5 a	2.4 a	15.3 a
New rotations:	VetchRye	Soya	70.5 a	7.5 ab	5.7 b	12.0 a	0.5 b	3.8 b
	Vetch	Soya	69.9 a	7.5 ab	6.3 b	10.3 a	0.6 b	5.5 b
	Fallow	Soya	70.7 a	7.2 ab	6.2 b	12.0 a	0.4 b	3.4 b
	VetchRye	Maize	69.2 a	3.3 c	10.4 a	13.2 a	0.6 b	3.3 b
	Vetch	Maize	71.2 a	6.0 b	7.4 b	12.2 a	0.3 b	2.9 b
	Fallow	Maize	72.9 a	8.5 a	6.0 b	9.2 a	0.6 b	2.6 b
One-way ANOVA (P-value): effects of the cropping system conversion								
Cropping system conversion			**0.01**	0.13	0.81	0.29	**<0.001**	**<0.001**
Two-way ANOVA (P-value): effects of cover crops and grain crops in new rotation systems								
Cover crop			0.58	**0.02**	**0.008**	0.53	0.86	0.28
Grain crop			0.64	**0.03**	**<0.001**	0.93	0.69	0.16
Cover crop × Grain crop			0.6	0.07	**<0.001**	0.36	0.05	0.41

Lowercase letters indicate significant differences among the crop rotation systems in volumes at *p* ≤ 0.05. Bold numbers indicate significant effects at *p* ≤ 0.05.

### Soil fungal functional guilds and the differential fungal genera

**Figure 3:**
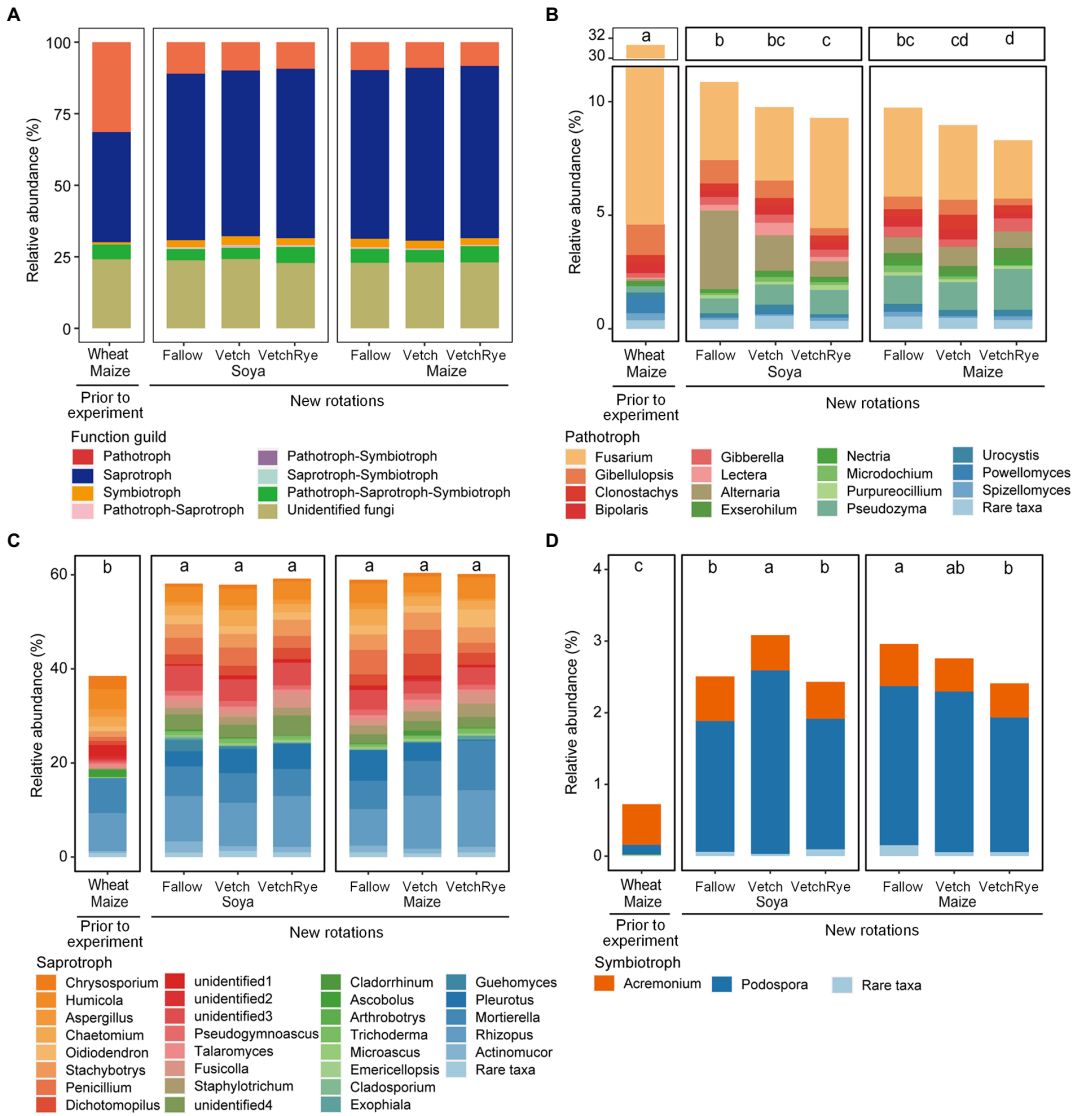
Relative abundances of fungal functional guilds **(A)** and the key fungal genera assigned to pathotrophs **(B)**, saprotrophs **(C)**, and symbiotrophs **(D)** in the new rotation systems compared to those present prior to the experiment. The rare genera that had relative abundances <0.1% are not included. Lowercase letters in the boxes indicate the significant differences among all cropping systems at *p* ≤ 0.05.

Soil fungal communitiy was assigned to pathotrophs, saprotrophs, symbiotrophs, and multi-functional fungi based on their functional guilds (Figure 3). The most fungi were assigned to saprotrophs, which accounted for 38.5 ~ 60.4%. Other fungal communities were assigned to pathotrophs and symbiotrophs, accounting for 8.3 ~ 31.4% and 0.7 ~ 3.1%, respectively. Multi-functional guilds (i.e., pathotroph–saprotroph, pathotroph–symbiotroph, saprotroph–symbiotroph and pathotroph–saprotroph–symbiotroph) accounted for 4.5 ~ 6.2% in total. The remaining 22.8 ~ 24.2% were unidentified fungi (Figure 3A).

The relative abundance of pathotrophs under the prior cropping system was relatively high, accounting for 31.4% (Figure 3B). Converting the wheat–maize system to the new rotation systems decreased 64.9 ~ 73.6% of the relative abundances of pathotrophs. Three differential pathogenic genera were identified by comparing the systems prior to and after the experiments. Specifically, compared with the prior rotation system, the abundances of *Fusarium*, *Powellomyces*, and *Gibellulopsis* were sharply decreased in the new rotation systems (Supplementary Table S4). Soya systems had more pathotrophs than the Maize systems, but the relative abundances of pathotrophs under these two systems followed the same order with respect to the cover crop: Fallow > Vetch > VetchRye (Figure 3B). The increase in pathotrophs under Soya were mainly contributed by the differential genera, including *Alternaria*, *Gibellulopsis*, and *Lectera* in Fallow, *Alternaria* and *Lectera* in Vetch, and *Lectera* and *Fusarium* in VetchRye (Supplementary Table S4). The differential pathogenic genera among cover crops showed that compared to Fallow, *Alternaria* and *Gibellulopsis* were decreased under Soya in Vetch and VetchRye, while *Gibellulopsis*, *Fusarium*, and *Microdochium* were decreased under Maize in Vetch and VetchRye.

The relative abundance of saprotrophs in the prior wheat–maize rotation system was 38.5% (Figure 3C). The new rotation systems increased 50.4 ~ 56.9% of the relative abundance of saprotrophs compared to that prior to the experiment, while the cover crop in the new rotation systems had no significant effect on saprotrophs. There were 12 differential saprotrophic genera between the wheat–maize and new rotation systems. The increase of these saprotrophic genera in new rotation systems accounted for 20.3–28.3% of the total abundances. Fot the new rotation systems, the changes in the saprotrophic genera were comparable for Soya and Maize; 26.3% vs. 26.6, 32.6% vs. 33.1, and 31.1% vs. 33.0% in Fallow, Vetch, and VetchRye, respectively (Supplementary Table S4).

**Figure 4:**
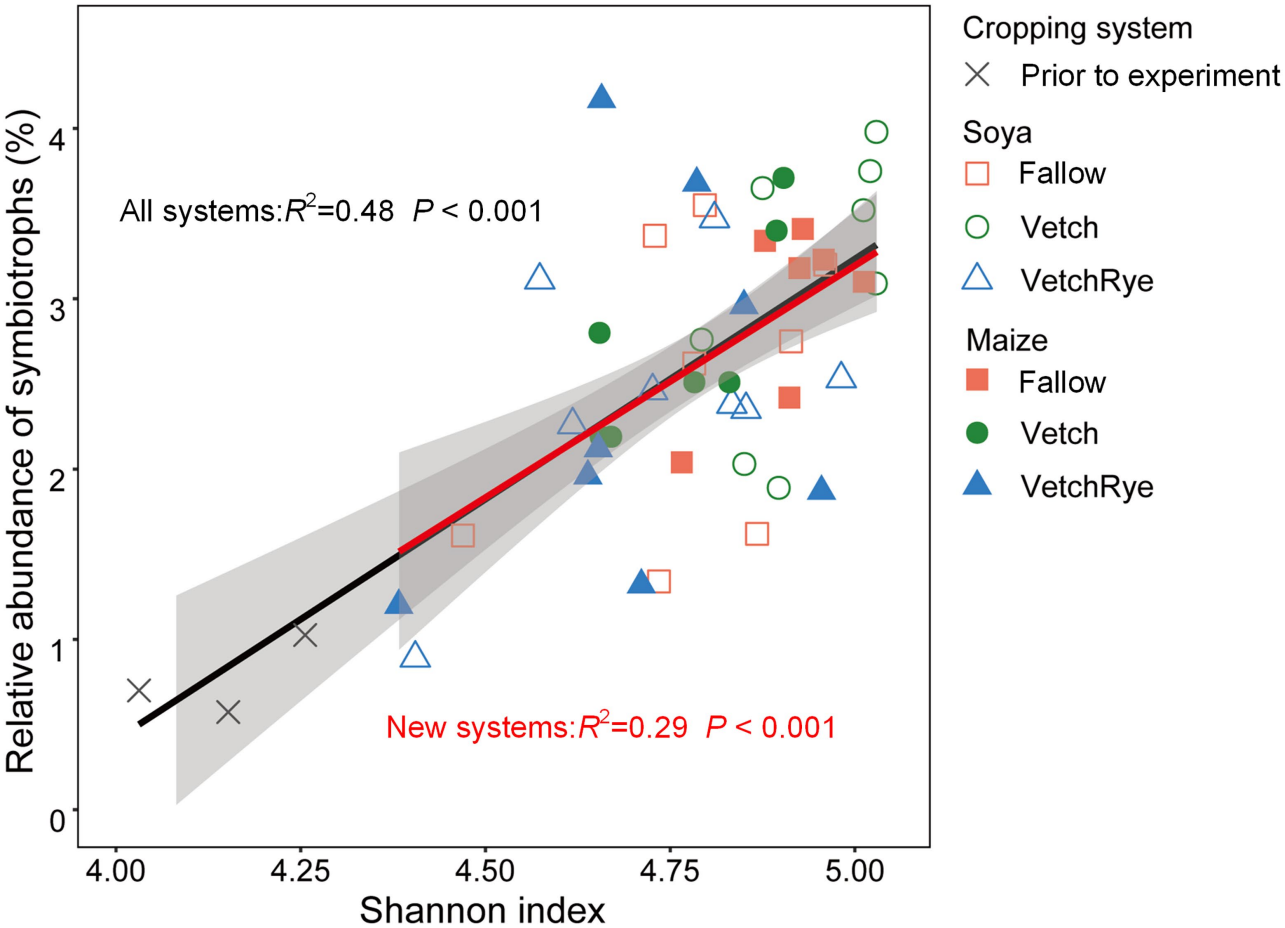
Correlations of the relative abundance of symbiotrophs with the Shannon index.

The relative abundance of symbiotrophs in the prior wheat–maize rotation system was 0.72% (Figure 3D). The relative abundance of the symbiotrophs increased by 2.3 ~ 3.3 fold in the new rotation systems after the experimental treatments were implemented. In Fallow, symbiotrophs were less under Soya than under Maize. Symbiotrophs were the most abundant in the Vetch–Soya system and the lowest in the VetchRye–Maize system. *Podospora* was the dominant differential symbiotrophic genus, which increased by 12.0 ~ 17.3 fold in the new rotation systems (Supplementary Table S4). The relative abundance of symbiotrophs was positively correlated with the Shannon Index (*p* ≤ 0.05), irrespective of system prior to and after the experiments (Figure 4).

### Environmental factors affecting fungal communities and guilds

**Figure 5:**
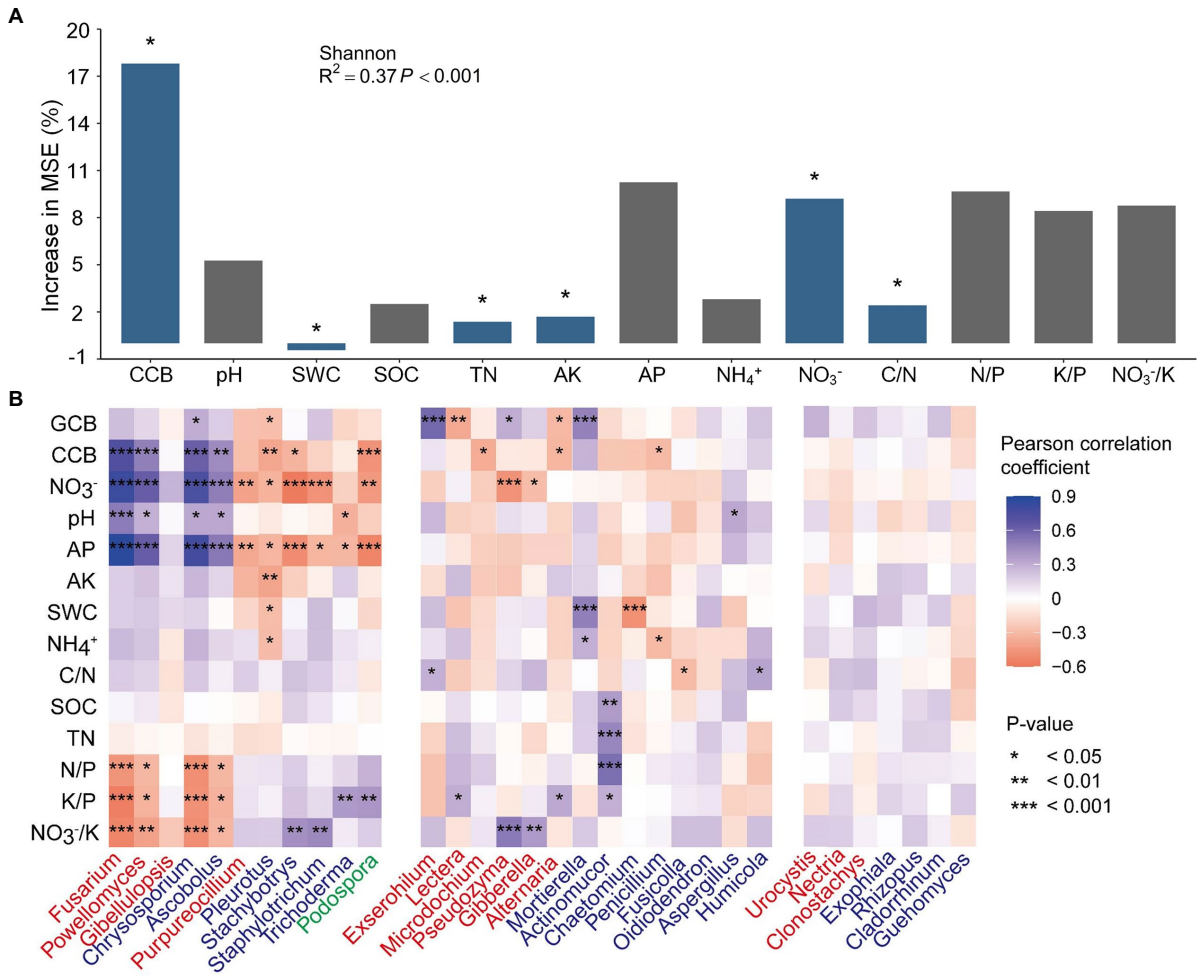
Random Forest mean predictor importance (% of increase in MSE) of the environmental factors as predictors of Shannon index **(A)**, and Pearson’s correlation coefficient between relative abundances of the differential fungal genera and environmental factors **(B)**. Blue or red color in the square indicates a positive or a negative correlation, respectively, and the shade of the color indicates the magnitude of the correlation coefficient. Fungal genera in red, blue, and green fonts represent the functional guilds: pathotrophs, saprotrophs, and symbiotrophs, respectively. Asterisks indicate the significance of the correlation at *p* ≤ 0.05 (*), 0.01 (**), or 0.001 (***). GCB, grain crop biomass; CCB, cover crop biomass; SWC, soil water content; SOC, soil organic carbon; TN, total nitrogen; AP, available phosphorus; AK, available potassium; C/N, the ratio of SOC and TN; N/P, the ratio of TN and AP; K/P, the ratio of AK and AP; NO_3_^−^/K, the ratio of soil nitrate and AK.

To identify the environmental factors (i.e., aboveground biomass and soil physicochemical properties) influencing fungal diversity and community composition, the primary controlling environmental factors were identified (Figure 5). The biomass of the cover crop, available P, NO_3_^−^, and soil stoichiometry were found to be important properties that affected the Shannon index, and the importance was significant for cover crop biomass, soil total nitrogen, available K, NO_3_^−^, and the C/N ratio (Figure 5A). For the functional guilds of the soil fungal community, cover crop biomass was significantly important for the relative abundance of pathotrophs, saprotrophs, and symbiotrophs; the C/N and K/P ratios were significantly important for the relative abundance of pathotrophs and saprotrophs; soil pH and available K was significant for pathotrophs, soil total nitrogen for saprotrophs, and NH_4_^+^ and NO_3_^−^/K for symbiotrophs (Supplementary Figure S4). The environmental factors were subsequently correlated to 32 differential genera (Figure 5B), defined in Supplementary Table S4, and accounted for 53.7 ~ 63.2% of the total fungal abundance. Soil NO_3_^−^, available P, K/P, and NO_3_^−^/K were found to be correlated with most differential genera. The pathotrophic *Fusarium* and *Powellomyces* and the saprotrophic *Chrysosporium* and *Ascobolus* were positively correlated with cover crop biomass, soil NO_3_^−^, pH, and available P, and negatively with N/P, K/P, and NO_3_^−^/K. The saprotrophic *Pleurotus*, *Stachybotrys* and *Staphylotrichum*, and the symbiotrophic *Podospora* were negatively correlated with cover crop biomass, soil NO_3_^−^, and available P. Some pathotrophs (i.e., *Exserohilum*, *Lectera*, *Microdochium*, *Pseudozyma*, and *Alternaria*) showed different correlation patterns from that of the most abundant pathogen *Fusarium*, but showed similar significant correlations with specific environmental factors to that of some saprotrophs and symbiotrophs (i.e., *Stachybotrys*, *Staphylotrichum*, *Trichoderma*, and *Podospora*), such as negative correlations with cover crop biomass and NO_3_^−^, and positive correlations with K/P and NO_3_^−^/K. The saprotrophic *Mortierella* was positively correlated with grain crop biomass, soil water content, and NH_4_^+^, while *Actinomucor* was positively correlated with SOC, TN, and the N/P and K/P ratios.

## Discussion

### Effects of the cropping system conversion

Consistent with our first hypothesis that if the prior wheat-maize system was changed to a new rotation system involving legumes, then soil fungal community would change along with the plant biomass and soil physicochemical properties, both the soil physicochemical properties and the fungal community were strongly affected by the change of the rotation system (Figures 1, 2; Table 1). In our study, the effects of changing the cropping system on the soil physicochemical properties and fungal community were similar to the effects of crop-type changes in previous long-term experiments (Ai et al., 2018; Azeem et al., 2020), which confirmed that the quick fungal response happened within 1 year was not a temporary change in this study. The new legume-involved rotation systems had lower soil pH, AK, AP, NO_3_^−^, and NH_4_^+^ concentrations than the prior system, mainly attributed to the cessation of mineral fertilization in new rotation systems. This is also supported by Xiang et al. (2020), who reported that mineral fertilization can reduce the soil pH and increase the content of available nutrients. Significant correlations between these soil properties and the soil fungal community composition indicated that changes in the soil nutrients could be the major controlling factor of fungal community composition between the prior and new systems (Figure 2A2). Moreover, the positive effects of the non-fertilized new rotation systems on the soil fungal diversity were in line with previous studies, which could be attributed to new niches provided by the nutrient-rich plant residue from the legumes (Ding et al., 2017; Thapa et al., 2021; Wang Y. et al., 2021). These results indicated that soil fungal response to the new rotation systems was as rapid as the changes in the soil nutrients.

The greater diversity of the soil fungi in new legume-involved rotation systems were linked to the altered soil physicochemical properties and the soil fungal guilds. Increases in the soil total nitrogen and decreases of available K and NO_3_^−^ were identified as the primary factors affecting fungal diversity (Figure 5A), indicating that adding leguminous cover crops and stopping mineral fertilization changed the soil nutrient status, which increased the fungal diversity. This is consistent with previous studies reporting that cover cropping increases fungal diversity, whereas high rates of fertilizer application reduce it (Tsiafouli et al., 2015; Schmidt et al., 2019). Moreover, increasing the number of crop species involved in a rotation system has been linked to increases in niche diversity, which could result in higher microbial diversity (Jesus et al., 2016). Increases in fungal diversity are often linked to shifts in the functional guilds of the fungal community, which was specifically indicated by the positive correlation between the Shannon index and symbiotrophs in our study (Figure 4). On the one hand, this is consistent with Jesus et al. (2016), who reported that increasing the number of crop species provided a greater diversity of plant hosts for symbiotrophs. On the other hand, the increase in oligotrophic symbiotrophs could result from the decrease in soil available N, P, and K (Table 1), because they have a growth advantage in relatively oligotrophic environments due to their interactions with plants (Reverchon et al., 2012). Additionally, the growth advantage of pathogens was negated when the abundance of symbiotrophs and saprotrophs increased (Figure 3), which was in line with the effects of cover crops reported in previous studies (Borrell et al., 2016; Schmidt et al., 2019).

Among the differential genera between the prior and new systems, the pathotrophic *Fusarium* and *Powellomyces*, which accounted for more than 20% of the relative abundance in the prior system, were significantly suppressed in the new systems. The abundant *Fusarium* in the prior system was consistent with a previous study that reported *Fusarium* accumulation in continuous wheat–maize systems (Landschoot et al., 2013). Most *Fusarium* species are fungal plant pathogens, and can produce mycotoxins in cereal crops and threaten public health through the food chain (Fones et al., 2020). A lower abundance of *Powellomyces* in the new systems could be an indication of increasing yield stability and sustainability, since the accumulation of *Powellomyces* can decrease plant shoot mass (Wang M. et al., 2021). By contrast, the enriched saprotrophic *Stachybotrys*, *Staphylotrichum*, *Penicillium*, and the symbiotrophic *Podospora* in the new systems have been identified as antagonists of soil-borne plant pathogens and the primary decomposers of organic matter in agricultural soils (Xu et al., 2012; Ding et al., 2017). Soil NO_3_^−^, available P and soil pH could be the factors controlling the abundance of differential genera, which were positively correlated with the pathotrophic *Fusarium* and *Powellomyces*, and negatively correlated with the saprotrophic *Pleurotus*, *Stachybotrys*, and *Staphylotrichum* and the symbiotrophic *Podospora* (Figure 5B). The correlation of these differential genera with the soil stoichiometry reflected the effects of the cover crop on fungal nutrient uptake strategies (Sadras, 2006; Adetunji et al., 2020).

Our results suggested that replacing mineral fertilizers with cover crops decreases N and P availability and substantially changes the sensitive microbial taxa. The role of the fungal community was changed from mineral nutrient uptake to organic matter decomposition by stimulating beneficial saprotrophs and symbiotrophs and suppressing pathogens in the new systems, which increased fungal diversity and improved soil and plant health.

### Different fungal responses to cover crops under succeeding maize and soya

Our second hypothesis that if cover crop is involved in maize and soya system, respectively, then the soil fungal community would respond to cover crop differently is accepted, because the diversity, composition, and functional guilds of soil fungal community showed different variation patterns under succeeding maize and soya (Figures 1–3). The legacy effects of the cover crop treatments were distinctly revealed when succeeded by maize but not soya (Figures 2B1,C1). Moreover, pairwise comparisons revealed significantly different soil fungal communities between maize and soya systems (Supplementary Table S2). On the one hand, this could be a result of altered soil properties and stoichiometry (Table 1) caused by different nutrient uptake preferences between soya and maize. Soya absorbs soil AP and AK, and decreases soil pH during biological N-fixation (Haynes, 1983), while maize tends to absorb soil NO_3_^−^ to satisfy its N demand (Gijsman, 1990; Fu et al., 2019). For example, the larger K/P ratio enriched *Alternaria* and *Lectera* in the soya systems, whereas the smaller K/P ratio and a lower soil NO_3_^−^ enriched *Pseudozyma* and *Exserohilum* in the maize systems (Figure 5). The different responses of these pathotrophic genera under maize and soya were consistent with Cannon et al. (2012) and Chamberlain et al. (2021). On the other hand, the different functional guilds in the soil fungal community could also be a result of the different root exudates between soya and maize that recruit specific microbial communities (Li et al., 2016; Hugoni et al., 2018). These results explained the different effects of cover crops in the maize and soya systems and confirmed that the microbial growth strategies were changed with different root activities and exploitation tactics in leguminous and cereal crops (Grayston et al., 1998).

In soya systems, cover crops significantly changed fungal diversity, despite that their effects on fungal community composition were overwhelmed by the strong effect of soya. Among the three cover crop treatments, Vetch had the highest fungal diversity (Figure 1). The Vetch–Soya rotation (a legume–legume succession) is an N-rich sequence due to N fixation of soya and nitrogen release from the vetch residues, which benefits fungi thriving (Di Lonardo et al., 2020). A legume–legume succession was also found to improve fungal diversity not only by increasing soil N, but also by recruiting more symbotrophs (Figure 4). Our results were consistent with the findings that legume crops enhanced the symbiotic relationships of symbiotrophic fungi due to shared developmental pathways between Rhizobium and fungal symbioses (Stracke et al., 2002; Turner et al., 2013). Although a legume–legume succession improved fungal diversity and symbiotrophic fungi, the changes in fungal community composition under soya systems were not directly due to changes in the cover crop biomass and soil nutrients (Figure 2B2). For example, the symbiotrophic *Podospora* showed the largest increase in the Vetch–Soya system, but it was not correlated with cover crop biomass and soil nutrients such as NH_4_^+^, NO_3_^−^, and available P (Figures 3D, 5B). The changes in *Podospora* and other fungal communities in a legume–legume succession are most likely due to root exudates from vetch and soya (Hugoni et al., 2018). Moreover, our results showed a Vetch–Soya system tended to decrease pathotrophs (Figure 3B), which is contrary to a study with continuous legume cropping (Strom et al., 2020). The accumulation of pathotrophs in a legume-legume succession may not have emerged yet within a year in this study. Another reason is that the root exudates and rhizosphere environment of these two legumes may be too different for the accumulation of their specific pathotrophs. However, further studies are still needed to explore the long-term effects of a Vetch-Soya rotation and the individual effects of vetch and soya on the fungal community. A long-term legume–legume succession may continuously enlarge the dominant group of microorganisms (Wang et al., 2020), and the rhizosphere environment is very likely different among legume species due to different rhizosheaths and root exudates (Kidd et al., 2018). Taken together, these results indicated that increasing the frequency of legume crops in rotation systems could increase soil fungal diversity and decrease the relative abundance of pathogens in the short term.

In maize systems, the cover crop had a significant effect on the fungal community, including its composition, diversity, and functions (Figures 1–3). Compared to fallow and single vetch, the mixed vetch and rye decreased the soil fungal diversity, which was consistent with a long-term study that reported a decrease in the Shannon index under mixed cover crops (Schmidt et al., 2019). Unlike the situation in soya systems, the fungal community composition in maize systems was affected by the cover crop biomass and soil nutrients (Figure 2C2). With an increase of the cover crop species, the increased biomass of the cover crops changed the soil chemical conditions, causing increased soil NH_4_^+^, and available P and K in this experiment (Adetunji et al., 2020; Zhang et al., 2021). The results indicated the differences in the cover crop biomass and soil nutrients were the main factors that shaped the soil fungal community composition under maize. These results were consistent with Acharya et al. (2019) and He et al. (2016), who also reported the importance of soil available nutrients with regard to the soil fungal community composition. Specifically, increasing the cover crop species in maize systems significantly decreased pathotrophs, possibly due to the recruitment of more saprotrophic *Mortierella* and *Staphylotrichum* in the mixed cover crop with more fresh substrates (Figures 3B,C). *Mortierella* is the first consumer of fresh substrates, and soil dominated by *Mortierella* showed strong suppression of the pathogenic *Fusarium* (Xiong et al., 2017; Li et al., 2020). The increases of *Mortierella* and *Staphylotrichum* and their positive correlations to soil NH_4_^+^, available P and the soil water content in a mixed cover crop treatment were in line with the findings of Allison et al. (2013), who reported that fungal saprotrophs were regulated by soil nutrient availability and moisture limitation. Moreover, the benefits of mixed vetch and rye in a maize system were greater than in a soya system (Figure 3B). These results indicated that litters of mixed vetch and rye litter in a maize system can provide appropriate soil nutrients and moisture for certain saprotrophs to grow, and those saprotrophs suppressed the growth of pathotrophs.

## Conclusion

This experiment confirmed the benefits of cropping systems that involved legumes and fallow on the soil fungal community, and the different responses of soil fungi to leguminous cover crops under soya and maize. Compared to a prior wheat–maize system, new rotation systems that replaced mineral fertilizer with cover crops changed soil fungal nutrient strategies, stimulated fungal diversity, as well as saprotrophs and symbiotrophs, and suppressed cereal-associated pathogens. Cover crop biomass and released nutrients significantly affected the soil fungal community composition in maize systems. But the effect of cover crops was overwhelmed by soya, and no cover crop effect were seen in soya systems. A legume–legume succession such as Vetch–Soya significantly increased the soil fungal diversity and symbiotrophs without stimulating legume-associated pathogens; however, a long-term study of a Vetch–Soya system is needed to eliminate the concern that potential pathogens may arise. The mixed cereal–legume cover crop produced the largest aboveground biomass, promoting the growth of particular saprotrophs. The proliferation of saprotrophs antagonized soil-borne pathogens, and the decrease of pathogens was larger under maize than soya systems. Overall, we provided evidence for the improvement of soil health by introducing legume-involved cropping systems due to changes in the soil fungal community.

## Data availability statement

The datasets presented in this study can be found in online repositories. The names of the repository/repositories and accession number(s) can be found at: https://db.cngb.org/search/project/, CNP0002196.

## Author contributions

SYu: formal analysis, data curation, writing–original draft, and visualization. TW: validation, writing–review and editing, and investigation. YM: writing–review and editing. SYa: conceptualization, project administration, and funding acquisition. LW: validation and investigation. HZ: formal analysis and data curation. YZ: resources. ZS: conceptualization, methodology, software, and validation. BZ: conceptualization, methodology, writing–review and editing, and project administration. All authors contributed to the article and approved the submitted version.

## Funding

This study was financially supported by the National Natural Science Foundation of China (No. 4207071613), National Key Research and Development Program of China (2021YFD1700200), and China Agriculture Research System (No. CARS-04).

## Conflict of interest

The authors declare that the research was conducted in the absence of any commercial or financial relationships that could be construed as a potential conflict of interest.

## Publisher’s note

All claims expressed in this article are solely those of the authors and do not necessarily represent those of their affiliated organizations, or those of the publisher, the editors and the reviewers. Any product that may be evaluated in this article, or claim that may be made by its manufacturer, is not guaranteed or endorsed by the publisher.

## Footnotes

^1^ https://www.r-project.org/

^2^ https://github.com/UMNFuN/FUNGuild

^3^ www.indexfungorum.org

^4^ https://github.com/pmartinezarbizu/pairwiseAdonis
