# Supplementary material for: Leguminous cover crops and soya increased soil fungal diversity and suppressed pathotrophs caused by continuous cereal cropping
Source: Front Microbiol. 2022 Oct 6;13:993214. doi: 10.3389/fmicb.2022.993214 (PMC9582142; doi:10.3389/fmicb.2022.993214)
Supplement: Supplementary file 1 [file Presentation_1.PPTX]

## Slide 1
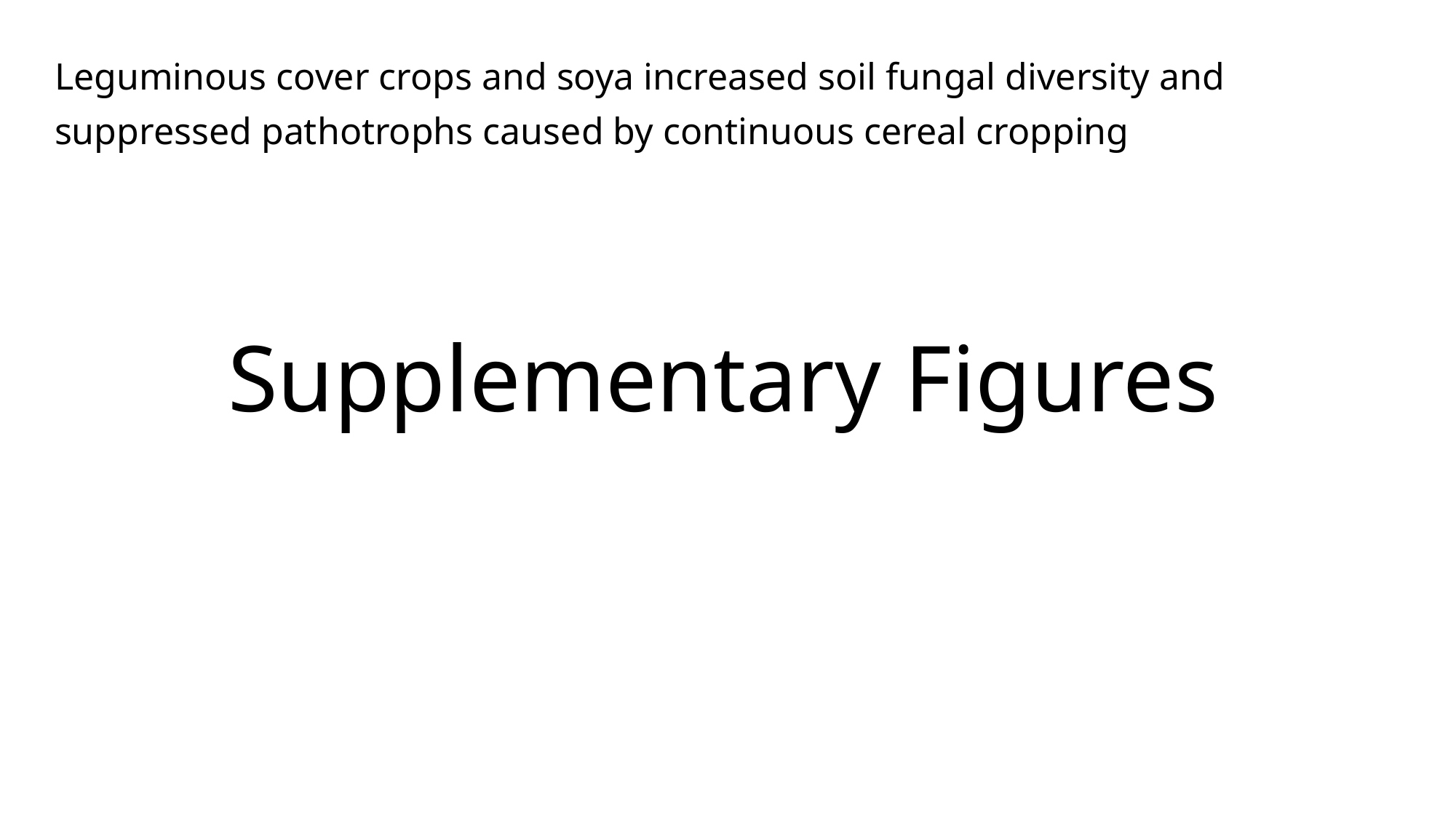

Leguminous cover crops and soya increased soil fungal diversity and
suppressed pathotrophs caused by continuous cereal cropping
# Supplementary Figures

## Slide 2
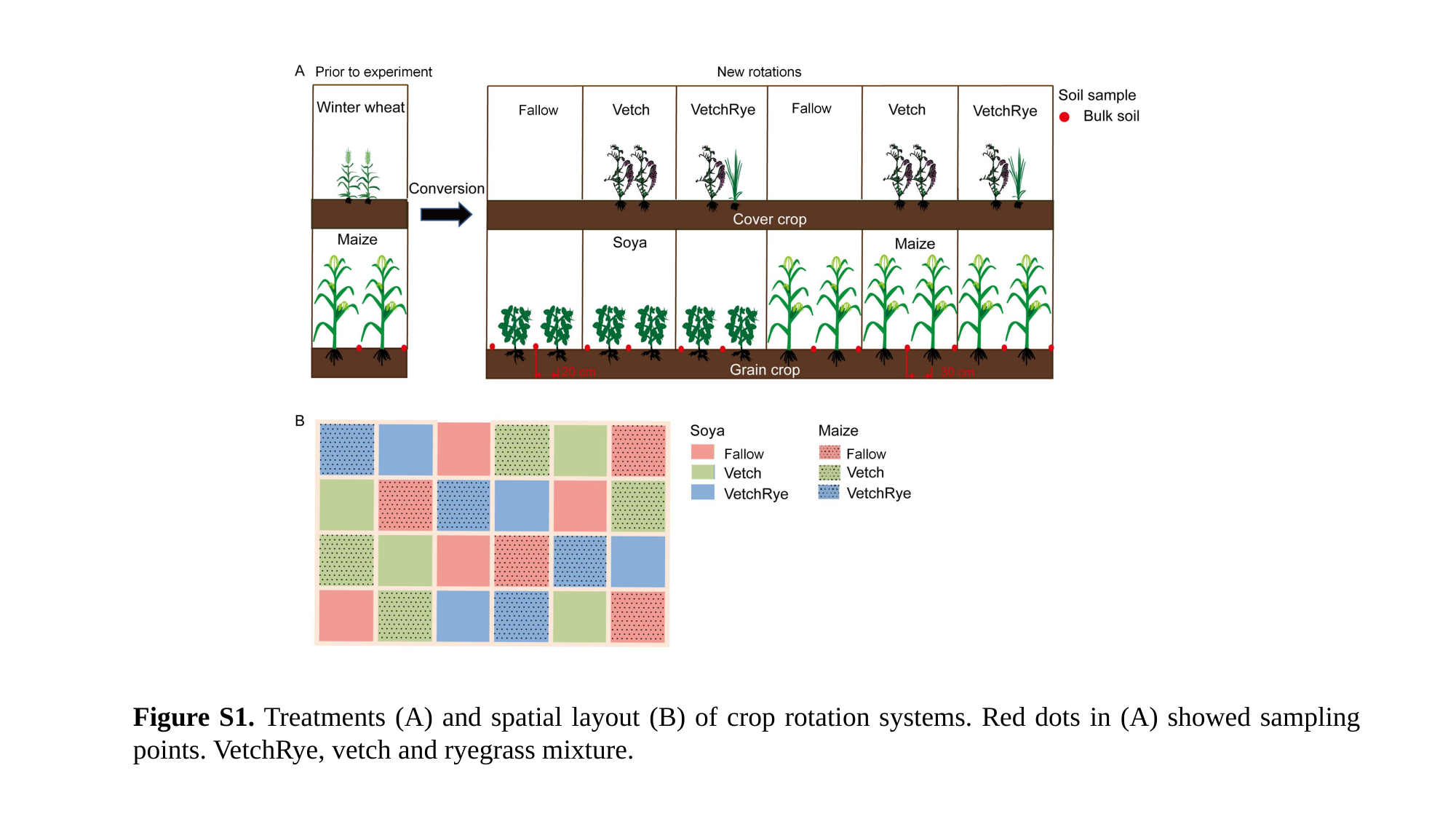

Figure S1. Treatments (A) and spatial layout (B) of crop rotation systems. Red dots in (A) showed sampling points. VetchRye, vetch and ryegrass mixture.

## Slide 3
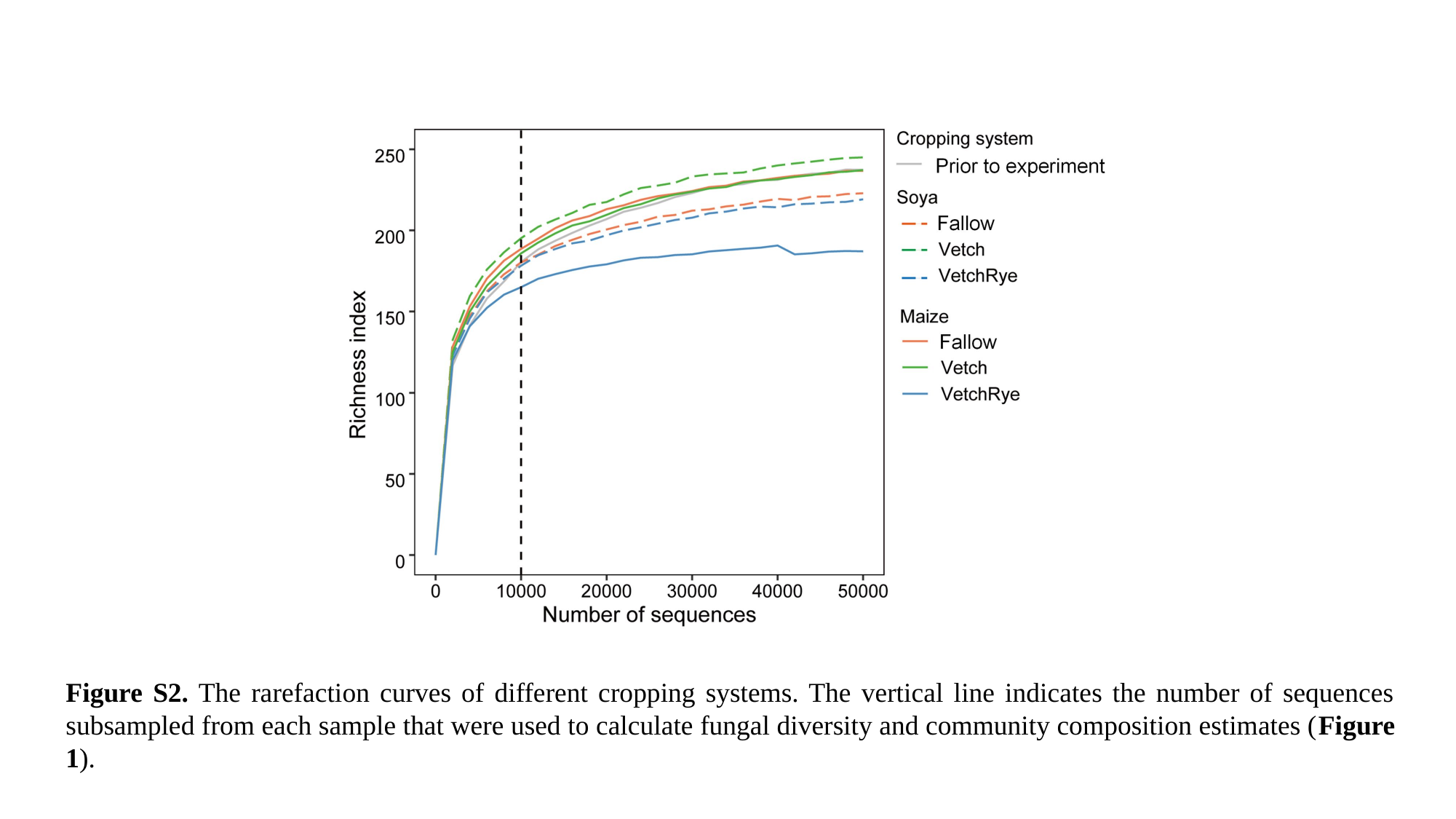

Figure S2. The rarefaction curves of different cropping systems. The vertical line indicates the number of sequences subsampled from each sample that were used to calculate fungal diversity and community composition estimates (Figure 1).

## Slide 4
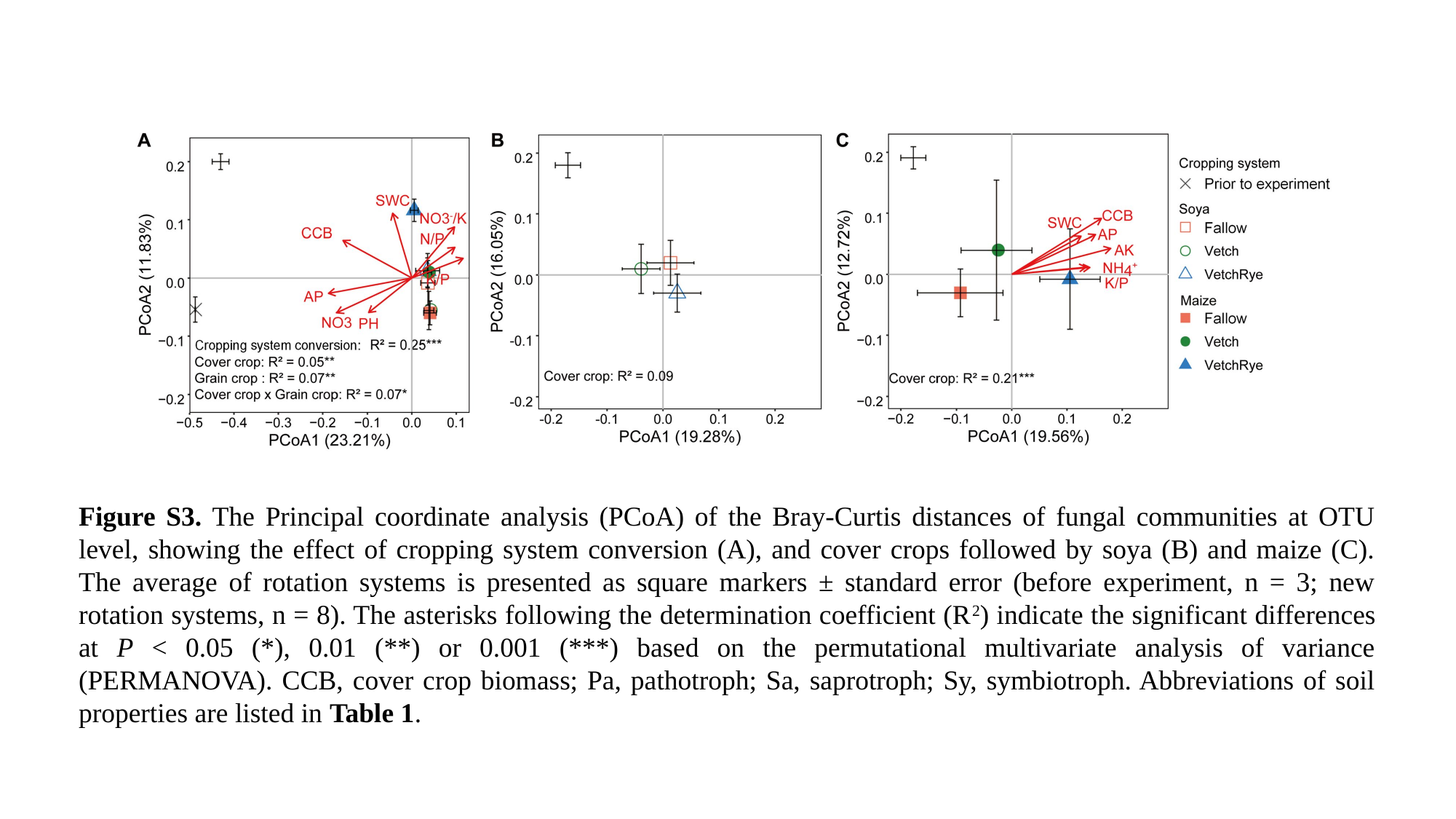

Figure S3. The Principal coordinate analysis (PCoA) of the Bray-Curtis distances of fungal communities at OTU level, showing the effect of cropping system conversion (A), and cover crops followed by soya (B) and maize (C). The average of rotation systems is presented as square markers ± standard error (before experiment, n = 3; new rotation systems, n = 8). The asterisks following the determination coefficient (R2) indicate the significant differences at P < 0.05 (*), 0.01 (**) or 0.001 (***) based on the permutational multivariate analysis of variance (PERMANOVA). CCB, cover crop biomass; Pa, pathotroph; Sa, saprotroph; Sy, symbiotroph. Abbreviations of soil properties are listed in Table 1.

## Slide 5
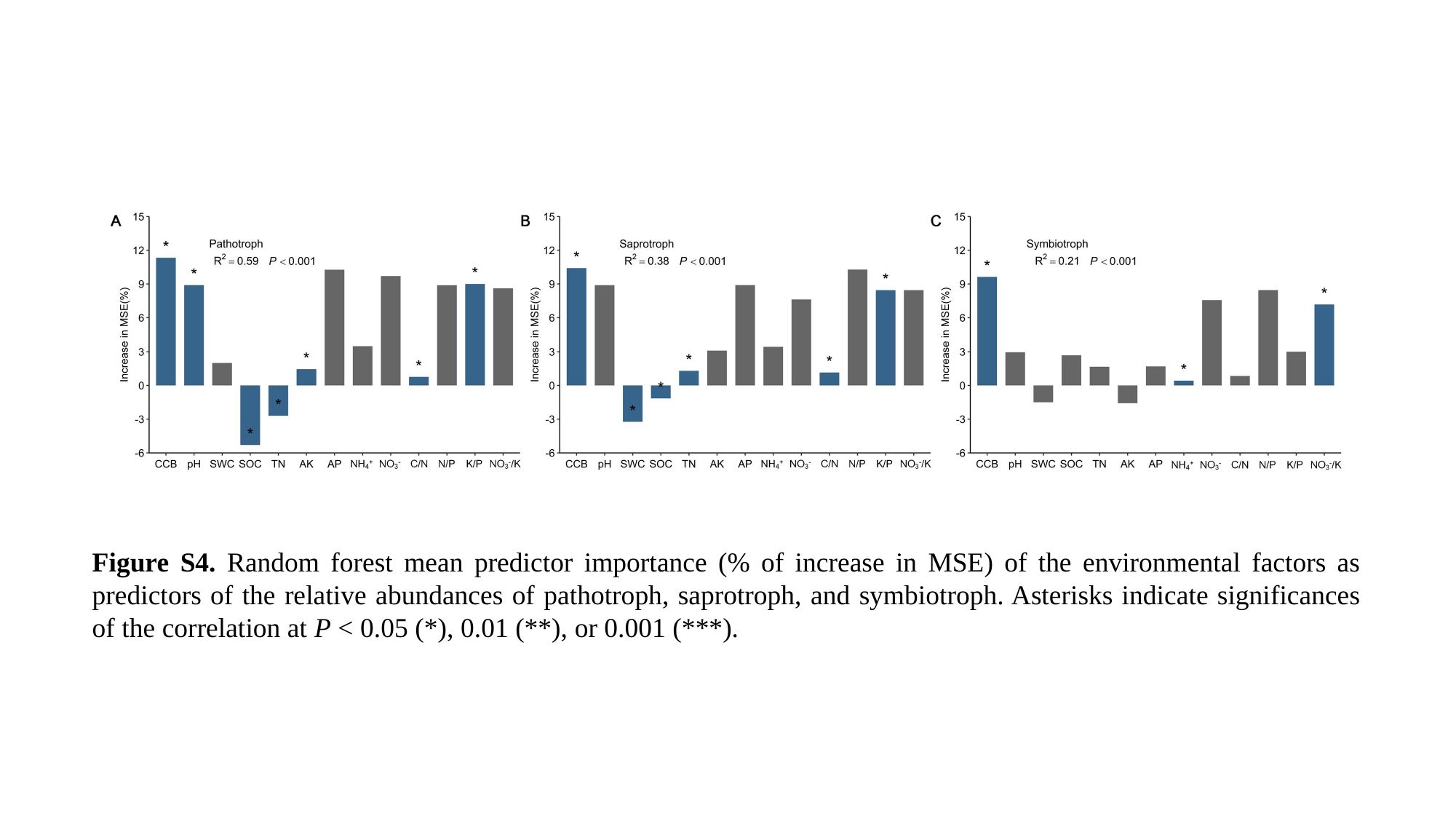

Figure S4. Random forest mean predictor importance (% of increase in MSE) of the environmental factors as predictors of the relative abundances of pathotroph, saprotroph, and symbiotroph. Asterisks indicate significances of the correlation at P < 0.05 (*), 0.01 (**), or 0.001 (***).
