# Supplementary material for: Leguminous cover crops and soya increased soil fungal diversity and suppressed pathotrophs caused by continuous cereal cropping
Source: Front Microbiol. 2022 Oct 6;13:993214. doi: 10.3389/fmicb.2022.993214 (PMC9582142; doi:10.3389/fmicb.2022.993214)
Supplement: Supplementary file 2 [file Data_Sheet_1.docx]

Supplementary Tables

## Table S1. The number of OTUs and genera assigned to eight functional guilds

| Functional guild | OTU No. | Genera No. |
| --- | --- | --- |
| Pathotroph | 131 | 56 |
| Saprotroph | 411 | 140 |
| Symbiotroph | 42 | 21 |
| Pathotroph-Saprotroph | 45 | 19 |
| Pathotroph-Symbiotroph | 2 | 2 |
| Saprotroph-Symbiotroph | 9 | 3 |
| Pathotroph-Saprotroph-Symbiotroph | 34 | 13 |
| Unidentified fungi | 126 | 33 |

## Table S2. Pairwise comparisons of soil fungal community composition at the genus level between each cropping system using Permutational multivariate analysis of variance (PERMANOVA)

| Paired cropping systems | | | F | R^2^ | *P* |
| --- | --- | --- | --- | --- | --- |
| Fallow-Soya | vs | Fallow-Maize | 1.76 | 0.119 | **0.037** |
| Vetch-Soya | vs | Vetch-Maize | 1.58 | 0.108 | **0.027** |
| VetchRye-Soya | vs | VetchRye-Maize | 2.75 | 0.164 | **0.002** |
| Fallow -Maize | vs | VetchRye-Maize | 4.37 | 0.252 | **0.001** |
| Vetch-Maize | vs | VetchRye-Maize | 2.69 | 0.171 | **0.002** |
| Fallow -Maize | vs | Vetch-Maize | 1.57 | 0.116 | 0.062 |
| Fallow -Soya | vs | Vetch-Soya | 0.82 | 0.055 | 0.604 |
| Fallow -Soya | vs | VetchRye-Soya | 1.19 | 0.078 | 0.261 |
| Vetch-Soya | vs | VetchRye-Soya | 1.47 | 0.095 | 0.142 |
| Fallow -Soya | vs | Vetch-Maize | 1.5 | 0.103 | 0.102 |
| Fallow -Soya | vs | VetchRye-Maize | 3.28 | 0.19 | **0.001** |
| Vetch-Soya | vs | Fallow -Maize | 1.13 | 0.08 | 0.313 |
| Vetch-Soya | vs | VetchRye-Maize | 4.43 | 0.24 | **0.002** |
| VetchRye-Soya | vs | Fallow -Maize | 1.7 | 0.116 | **0.029** |
| VetchRye-Soya | vs | Vetch-Maize | 1.38 | 0.096 | 0.143 |

Statistical significance showed in bold (*P* < 0.05)

## Table S3. Correlation of cover crop biomass and soil properties with the PCoA variances of fungal community composition at the genus level prior to and after experiment, and among cover crop treatments in maize systems according to Permutational multivariate analysis of variance (PERMANOVA)

| Factors | Prior to and after the  experiment | | | |  | Among cover crops  in maize systems | | | |
| --- | --- | --- | --- | --- | --- | --- | --- | --- | --- |
|  | PCoA1 | PCoA2 | R^2^ | *P* |  | PCoA1 | PCoA2 | R^2^ | *P* |
| CCB | -0.90 | 0.43 | 0.66 | **0.001** |  | 0.97 | -0.23 | 0.51 | **0.003** |
| SWC | -0.34 | 0.94 | 0.16 | **0.016** |  | 0.98 | -0.21 | 0.25 | **0.049** |
| NH_4_^+^ | -0.78 | 0.63 | 0.12 | 0.064 |  | 0.97 | -0.26 | 0.31 | **0.033** |
| NO_3_^-^ | -1.00 | -0.10 | 0.75 | **0.001** |  | 0.99 | -0.14 | 0.03 | 0.761 |
| pH | -0.98 | -0.18 | 0.21 | **0.005** |  | -0.99 | 0.15 | 0.20 | 0.117 |
| TN | 0.01 | 1.00 | 0.04 | 0.405 |  | 0.84 | 0.54 | 0.05 | 0.604 |
| SOC | -0.39 | 0.92 | 0.03 | 0.535 |  | 0.51 | 0.86 | 0.03 | 0.758 |
| C/N | -0.85 | -0.53 | 0.02 | 0.655 |  | -0.99 | 0.12 | 0.02 | 0.829 |
| AK | -0.85 | 0.53 | 0.12 | **0.045** |  | 0.94 | -0.35 | 0.56 | **0.001** |
| AP | -1.00 | 0.07 | 0.86 | **0.001** |  | 0.97 | -0.26 | 0.43 | **0.004** |
| N/P | 0.98 | 0.18 | 0.23 | **0.005** |  | -0.47 | 0.89 | 0.04 | 0.715 |
| NO_3_^-^/K | 0.91 | 0.42 | 0.32 | **0.001** |  | 0.98 | -0.18 | 0.06 | 0.535 |
| K/P | 1.00 | -0.03 | 0.30 | **0.001** |  | 0.92 | -0.39 | 0.30 | **0.026** |

CCB, cover crop biomass; SWC, soil water content; SOC, soil organic carbon; TN, total nitrogen; AP, available phosphorus; AK, available potassium; C/N, the ratio of SOC and TN; N/P, the ratio of TN and AP; NO_3_^-^/K, the ratio of soil nitrate and AK; K/P, the ratio of AK and AP.

## Table S4. Relative abundances of general fungal genera assigned to pathotrophs, saprotrophs and symbiotrophs in the systems prior to and after the experiment

| Function  guild | Phylum | Genus | Prior to  exper-  ment |  | New rotation systems | | | | | | |
| --- | --- | --- | --- | --- | --- | --- | --- | --- | --- | --- | --- |
|  |  |  |  |  | Soya | | |  | Maize | | |
|  |  |  |  |  | Fallow | Vetch | VetchRye |  | Fallow | Vetch | VetchRye |
| Patho-  trophs | Ascomycota | *Fusarium* | 26.81 a |  | 3.44 b | 3.23 b | 4.85 b |  | 3.92 b | 3.29 b | 2.56 c |
|  | Ascomycota | *Gibellulopsis* | 1.35 a |  | 1.03 b | 0.78 bc | 0.32 d |  | 0.55 c | 0.66 c | 0.3 d |
|  | Ascomycota | *Clonostachys* | 0.35 b |  | 0.33 b | 0.34 b | 0.31 b |  | 0.33 b | 0.64 a | 0.33 b |
|  | Ascomycota | *Bipolaris* | 0.44 a |  | 0.27 a | 0.39 a | 0.31 a |  | 0.44 a | 0.45 a | 0.24 a |
|  | Ascomycota | *Gibberella* | 0.2 c |  | 0.35 b | 0.36 b | 0.32 b |  | 0.46 ab | 0.33 b | 0.57 a |
|  | Ascomycota | *Lectera* | 0.06 c |  | 0.25 b | 0.55 a | 0.19 b |  | 0 d | 0 d | 0 d |
|  | Ascomycota | *Alternaria* | 0.08 d |  | 3.46 a | 1.56 b | 0.68 c |  | 0.7 c | 0.84 c | 0.74 c |
|  | Ascomycota | *Exserohilum* | 0.16 bcd |  | 0.04 d | 0.09 d | 0.11 cd |  | 0.37 ab | 0.36 abc | 0.52 a |
|  | Ascomycota | *Nectria* | 0.07 b |  | 0.13 ab | 0.18 ab | 0.12 ab |  | 0.19 ab | 0.11 b | 0.22 a |
|  | Ascomycota | *Microdochium* | 0.01 c |  | 0.1 b | 0.21 ab | 0.14 b |  | 0.29 a | 0.13 b | 0.05 bc |
|  | Ascomycota | *Purpureocillium* | 0 c |  | 0.14 ab | 0.12 b | 0.22 a |  | 0.15 ab | 0.12 b | 0.12 b |
|  | Basidiomycota | *Pseudozyma* | 0.27 e |  | 0.66 d | 0.89 cd | 1.07 bc |  | 1.24 b | 1.23 b | 1.8 a |
|  | Basidiomycota | *Urocystis* | 0.2 abc |  | 0.14 bc | 0.25 a | 0.11 c |  | 0.23 ab | 0.24 ab | 0.24 a |
|  | Chytridiomycota | *Powellomyces* | 0.71 a |  | 0.05 b | 0.17 b | 0.03 b |  | 0.12 b | 0.03 b | 0.04 b |
|  | Chytridiomycota | *Spizellomyces* | 0.31 a |  | 0.09 a | 0.07 a | 0.14 a |  | 0.2 a | 0.07 a | 0.16 a |
|  | Rare taxa |  | 0.38 a |  | 0.4 a | 0.57 a | 0.36 a |  | 0.55 a | 0.49 a | 0.39 a |
| Sapro-  troph | Ascomycota | *Chrysosporium* | 2.79 a |  | 0.59 b | 0.87 b | 0.56 b |  | 0.75 b | 0.75 b | 0.7 b |
|  | Ascomycota | *Humicola* | 4.3 ab |  | 3.33 b | 3.52 ab | 3.9 ab |  | 4.23 ab | 3.39 b | 4.5 a |
|  | Ascomycota | *Aspergillus* | 1.59 a |  | 0.77 b | 0.99 b | 0.94 b |  | 1.28 ab | 0.85 b | 0.48 c |
|  | Ascomycota | *Chaetomium* | 2.05 b |  | 1.96 b | 3.43 a | 1.73 b |  | 3.4 a | 2.01 b | 1.84 b |
|  | Ascomycota | *Oidiodendron* | 1.06 c |  | 1.98 b | 1.72 b | 1.64 b |  | 1.96 b | 1.46 bc | 3.83 a |
|  | Ascomycota | *Stachybotrys* | 1.14 b |  | 2.89 a | 2.87 a | 3.42 a |  | 3.29 a | 3.64 a | 3.29 a |
|  | Ascomycota | *Penicillium* | 0.89 d |  | 3.53 b | 3.85 b | 2.52 c |  | 5.19 a | 5.1 a | 2.12 c |
|  | Ascomycota | *unidentified1* | 2.38 a |  | 0.37 b | 0.84 b | 0.76 b |  | 0.93 b | 0.82 b | 0.55 b |
|  | Ascomycota | *unidentified2* | 0.71 a |  | 0.02 c | 0.04 bc | 0.02 bc |  | 0.03 bc | 0.42 b | 0.03 bc |
|  | Ascomycota | *unidentified3* | 0.42 d |  | 5.29 a | 4.57 ab | 4.81 ab |  | 4.19 ab | 2.58 c | 3.68 b |
|  | Ascomycota | *Talaromyces* | 0.72 a |  | 1.12 a | 0.89 a | 0.81 a |  | 0.88 a | 1.22 a | 0.62 a |
|  | Ascomycota | *Fusicolla* | 0.25 c |  | 1.56 b | 1.37 b | 3.01 a |  | 1.35 b | 1.28 b | 2.37 ab |
|  | Ascomycota | *Staphylotrichum* | 0.2 c |  | 1.39 b | 1.62 b | 1.68 b |  | 1.88 b | 2.05 ab | 2.84 a |
|  | Ascomycota | *unidentified4* | 0.15 d |  | 3.24 ab | 2.71 b | 4.24 a |  | 1.88 c | 2.05 bc | 2.24 b |
|  | Ascomycota | *Cladorrhinum* | 0.07 c |  | 0.25 b | 0.07 c | 0.01 c |  | 0.12 c | 0.98 a | 0.08 c |
|  | Ascomycota | *Ascobolus* | 1.4 a |  | 0 c | 0.09 b | 0 c |  | 0.02 b | 0 c | 0 c |
|  | Ascomycota | *Arthrobotrys* | 0.06 a |  | 0.12 a | 0.11 a | 0.18 a |  | 0.11 a | 0.12 a | 0.22 a |
|  | Ascomycota | *Trichoderma* | 0.08 c |  | 0.78 ab | 0.92 a | 0.69 b |  | 0.51 b | 0.6 b | 0.95 a |
|  | Ascomycota | *Microascus* | 0.05 b |  | 0.13 b | 0.3 ab | 0.41 ab |  | 0.45 ab | 0.48 a | 0.3 ab |
|  | Ascomycota | *Emericellopsis* | 0.04 a |  | 0.13 a | 0.11 a | 0.13 a |  | 0.07 a | 0.1 a | 0.15 a |
|  | Ascomycota | *Cladosporium* | 0.04 b |  | 0.37 a | 0.17 ab | 0.11 b |  | 0.06 b | 0.04 b | 0.05 b |
|  | Ascomycota | *Exophiala* | 0.01 b |  | 0.38 ab | 0.1 b | 0.13 ab |  | 0.07 b | 0.13 ab | 0.76 a |
|  | Basidiomycota | *Guehomyces* | 0.03 c |  | 2.39 a | 0.6 b | 0.17 b |  | 0.11 b | 0.2 b | 0.18 b |
|  | Basidiomycota | *Pleurotus* | 0 d |  | 3.24 b | 5.1 a | 5.29 a |  | 6.42 a | 3.77 b | 0.14 c |
|  | Mortierellomycota | *Mortierella* | 7.39 b |  | 6.27 c | 6.32 c | 5.71 c |  | 6.07 c | 7.41 b | 10.52 a |
|  | Mucoromycota | *Rhizopus* | 8.11 cd |  | 9.67 c | 9.2 c | 10.88 b |  | 7.77 d | 11.24 ab | 12.02 a |
|  | Mucoromycota | *Actinomucor* | 0.4 c |  | 2.35 a | 1.06 b | 1.15 b |  | 1.4 b | 1 b | 1.18 b |
|  | Ascomycota | *Dichotomopilus* | 0.88 c |  | 2.04 b | 2.04 b | 2.39 b |  | 2.38 ab | 4.63 a | 2.51 ab |
|  | Ascomycota | *Pseudogymnoascus* | 0.44 c |  | 0.99 b | 1.18 ab | 0.91 b |  | 1.14 ab | 1.32 a | 1.02 ab |
|  | Rare taxa |  | 0.85 ab |  | 0.96 ab | 1.23 a | 0.98 ab |  | 0.98 ab | 0.76 b | 0.97 ab |
| Symbio  -troph | Ascomycota | *Acremonium* | 0.57 a |  | 0.62 a | 0.49 a | 0.52 a |  | 0.59 a | 0.46 a | 0.48 a |
|  | Ascomycota | *Podospora* | 0.14 c |  | 1.82 b | 2.56 a | 1.82 b |  | 2.22 ab | 2.24 ab | 1.88 ab |
|  | Rare taxa |  | 0.02 b |  | 0.06 b | 0.03 b | 0.09 ab |  | 0.15 a | 0.06 b | 0.06 b |

Rare taxa indicate the fungal genera had a relative abundance lower than 0.1%.

## Table S5. Random forest mean predictor importance (% of increase in MSE) of environmental factors as predictors for Richness index.

| Environmental factor | Increase in MSE (%) | *P* |
| --- | --- | --- |
| CCB | 7.5 | 0.08 |
| pH | 1.61 | 0.36 |
| SWC | -0.99 | 0.66 |
| SOC | 6.26 | 0.06 |
| TN | 1.98 | 0.38 |
| AK | -1.32 | 0.81 |
| AP | 7.71 | 0.06 |
| NH_4_^+^ | 1.9 | 0.37 |
| NO_3_^-^ | 4.02 | 0.22 |
| C/N | -2.59 | 0.88 |
| N/P | 2.75 | 0.38 |
| K/P | 2.37 | 0.54 |
| NO_3_^-^/K | 3.89 | 0.24 |
| Full Model: R^2^ = 4.3 %, *P* = 0.33 | | |

Significance of the models and cross-validated R^2^ values were assessed with 5000 permutations of the Richness index. Statistical significance showed in bold (*P* < 0.05).
